# Papers on Cholera

**Published:** 1831

**Authors:** 


Papers on Cholera. By the Board of Health. Octavo, pp. 38. Aug. 1831.

The greater part of this brochure is occupied with some brief abstract of the symptoms and treatment of the Indian cholera; but this we need not notice, as the pages of our journal contain much ampler information on these points than can be conveyed in a pamphlet.

The next document is an extract from Dr. Keir's report on the cholera of Moscow, as it appeared there in the

Autumn of 1830, and Winter of 1831. As we shall quote the description of the symptoms from Drs. Russell and Barry, we shall here only insert Dr. Keir's *post mortem* appearances.

"The appearances in the dead bodies were not uniform, and varied according to the duration of the disease and the circumstances under which the patient had died. As this was the case, I conceive the most satisfactory way by which I can answer the inquiry on this head will be to transmit the printed accounts of the dissections made at Moscow, and presented to the Medical Council there, by its members who occupied themselves the most with this part of the duty; while I here add the impression made on my own mind by the dissections at which I was present.

The extremities in general were more or less livid and contracted, and the skin of the hands and feet corrugated, the features sunk and ghastly; on opening the skull, the blood-vessels of the brain and its membranes were more or less turgid with blood, particularly towards the base; the arachnoidea had sometimes in several places lost its transparency, and adhered to the pia mater; a fluid was sometimes found effused into the convolutions of the brain in some quantity, and more or less of serum in the lateral ventricles. The blood-vessels of the vertebral column and spinal chord more or less loaded with blood, which was sometimes effused between its arachnoid and dura mater; partial softening of the substance of the spinal chord was sometimes met with, and marks of inflammatory congestion in the larger nerves. The lungs were generally gorged with dark-coloured blood, the cavities of the heart filled with the same, and frequently containing polypous concretions. In all the dissections I was present at, very dark-coloured blood, which, when extended on a white surface, resembled the colour of the darkest cherry, was found in the arch of the aorta, and in other arteries.

The state of the abdominal organs varied considerably, the stomach and different parts of the intestines were frequently found to be partially, but considerably contracted; the internal surface of the stomach sometimes seemed to be little affected. A whitish or yellow fluid matter resembling the evacuations was frequently found in different parts of the alimentary canal, which now and then contained a good deal of air. In either case, both stomach and intestines bore marks of congestion, and of a sub-inflammatory state, varying from dark-coloured spots of small extent, to several inches, affecting the whole internal circumference of the intestines. The colour of these parts also varied a good deal, from dark-coloured venous congestion, to rose-coloured inflammation. In one case the internal surface of the stomach was so strongly and so generally tinged of a very dark colour, that it might easily have been mistaken for gangrene. On exposing the stomach between the eye and the light, it was evident that there was neither gangrene, nor solution of continuity, but that the dark colour proceeded from a very general and great congestion of very dark-coloured blood in the vessels of the organ. The subject of this case, I was told, had died with symptoms of a typhoid nature, after suffering from the usual symptoms of the epidemic. Excepting in this case, which was evidently one of congestion, and not of inflammation, I saw nothing in the morbid appearances from which a conclusion could be drawn, that inflammation was a very general morbid change in the alimentary canal, or a common cause of death: however, by its presence in the second period of the disease, it might add to the general irritation, or, even as a consequence of preceding congestion, be itself occasionally the cause of the fatal event. Both stomach and bowels were frequently of a paler colour than natural, both on the outer and inner surface; but neither thickening nor condensation from inflammation, nor exulceration, destruction of substance, nor abscess, appeared in any of the dissections I was present at.

The liver was generally pretty full of dark-coloured blood, the gall-bladder frequently much distended with tenacious ropy bile, of a dark yellow or green colour; the gall-ducts sometimes contracted, at other times not; the appearance of the pancreas, spleen, and kidneys was various, frequently differing but little from their natural state, in other cases rather surcharged with blood, the urinary bladder almost always collapsed and empty, the uterus generally natural."

The last document, and that which will be most eagerly perused, is an extract from the joint report of Drs. Russell and Barry. We shall give it entire.

"*St. Petersburgh, July* 27, 1831.

Sir,

Although there can be no doubt that the disease now prevailing here is strictly identical, in all essential points, with the Epidemic Cholera of India; and although there are many descriptions extant of that malady, much more ably and accurately drawn up than any which we can pretend to give; yet we are induced to believe that a short account of the symptoms which we ourselves have actually witnessed and noted at the bedside in some hundreds of cases, since our arrival here, may be useful,—first, because we are not aware that any description by an eye-witness of European Cholera has yet been addressed to the British Government; secondly, because the disease, as it has shown itself in this capital, when closely compared with the Indian Cholera, appears to have undergone some modifications; thirdly, because, having now studied the disease in all its stages, our description, however imperfect, will at least assist towards establishing a standard of comparison with other local epidemics of Cholera in Europe, and may, perhaps, enable those who have not seen this disease, to recognise it with more certainty than they would otherwise be able to do.

The Cholera Morbus of the North of Europe, to which the Russian peasants have given the name of 'Chornaia Colezn,' or *black illness*, like most other diseases, is accompanied by a set of symptoms which may be termed preliminary; by another set which strongly mark the disease in its first, cold, or collapse stage; and by a third set, which characterise the second stage, that of reaction, heat, and fever.

*Preliminary Symptoms.* We have but few opportunities of witnessing the presence of all these symptoms, some of which precede the complete seizure by so short an interval, that the utmost diligence is scarcely sufficient to bring the patient and the physician together, after their occurreuce, before the disease is fully formed. Diarrhœa, at first feculent, with slight cramps in the legs, nausea, pain, or heat about the pit of the stomach, malaise, give the longest warning. Indeed, purging, or ordinary diarrhœa, has been frequently known to continue for one, two, or more days, unaccompanied by any other remarkable symptom, until the patient is suddenly struck blue, and nearly lifeless. Often the symptoms just mentioned are arrested by timely judicious treatment, and the disease completely averted. When violent vertigo, sick stomach, nervous agitation, intermittent, slow, or small pulse, cramps, beginning at the tips of the fingers and toes, and rapidly approaching the trunk, give the first warning; then there is scarcely an interval. Vomiting or purging, or both these evacuations, of a liquid like rice-water or whey, or barley-water, come on; the features become sharp and contracted, the eye sinks, the look is expressive of terror, wildness, and, as it were, a consciousness on the part of the sufferer that the hand of death is upon him. The lips, the face, the neck, the hands, the feet, and soon the thighs, arms, and whole surface, assume a leaden, blue, purple, black, or deep brown tint, according to the complexion of the individual, varying in shade with the intensity of the attack. The fingers and toes are reduced at least a third in thickness; the skin and soft parts covering them are wrinkled, shrivelled, and folded; the nails put on a blueish pearl-white; the larger superficial veins are marked by flat lines of a deeper black; the pulse is either small as a thread, and scarcely vibrating, or else totally extinct. The skin is deadly cold, and often damp; the tongue *always*

*moist*, often white and loaded, but flabby and chilled, like a bit of dead flesh. The voice is nearly gone; the respiration quick, irregular, and imperfectly performed. Inspiration appears to be effected by an immense effort of the chest, whilst the alæ nasi (in the most hopeless cases, and towards their close), instead of expanding, collapse, and stop the ingress of the air. Expiration is quick and convulsive. The patient asks only for water, speaks in a plaintive whisper (the 'vox cholerica'), and only by a word at a time, from not being able to retain air enough in his lungs for a sentence. He tosses incessantly from side to side, and complains of intolerable weight and anguish around his heart. He struggles for breath, and often lays his hand on his stomach and chest to point out the seat of his agony. The integuments of the belly are sometimes raised into high irregular folds, whilst the belly itself is violently drawn in, the diaphragm upwards and inwards towards the chest: sometimes there are tetanic spasms of the legs, thighs, and loins; but we have not seen general tetanus, nor even trismus. There is occasionally a low, suffering whine. The secretion of urine is always totally suspended, nor have we observed tears shed under these circumstances; vomiting and purging, which are far from being the most important or dangerous symptoms, and which, in a very great number of cases of the present epidemic have not been profuse, generally cease, or are arrested by medicine easily in the attack. Frictions remove the blue colour for a time from the part rubbed; but in other parts, particularly the face, the livor becomes every moment more intense and more general. The lips and cheeks sometimes puff out and flap, in expiration, with a white froth between them, as in apoplexy. If blood be obtained in this state, it is black, flows by drops, is thick, and feels to the finger colder than natural. Towards the close of this scene, the respiration becomes very slow, there is a quivering among the tendons of the wrist, the mind remains entire. The patient is first unable to swallow, then becomes insensible; there never is, however, any rattle in the throat, and he dies quietly after a long, convulsive sob or two.

The above is a faint description of the very worst kind of case, dying, in the cold stage, in from six to twenty-four hours after the setting in of the bad symptoms. We have seen many such cases just carried to the hospital from their homes or their barracks. In by far the greater number vomiting had ceased, in some, however, it was still going on, and invariably of the true serous kind. Many confessed that they had concealed a diarrhœa for a day or two; others had been suddenly seized, generally very early in the morning.

From the aggravated state which we have just described, but very few indeed recover, particularly if that state has been present even *for four hours* before treatment has commenced. A thread of pulse, however small, is almost always felt at the wrist, where recovery from the blue or cold stage is to be expected. Singular enough to say, hiccough coming on in the intermediate moments, between the threatening of death and the beginning of reaction, is a favourable sign, and generally announces the return of circulation.

In less severe cases, the pulse is not wholly extinguished, though much reduced in volume; the respiration is less embarrassed; the oppression and anguish at the chest are not so overwhelming, although vomiting and purging and the cramps may have been more intense. The coldness and change of colour of the surface, the peculiar alteration of the voice, a greater or less degree of coldness of the tongue, the character of the liquids evacuated, have been invariably well marked in all the degrees of violence of attack which we have hitherto witnessed in this epidemic. In no case or stage of this disease have we observed shivering; nor have we heard, after inquiry, of more than one case, in which this febrile symptom took place.

*Fever or Hot Stage.* After the blue cold period has lasted from twelve to twenty-four, seldom to forty-eight hours or upwards, the pulse and external heat begin gradually to return, head-ache is complained of, with noise in the ears, the tongue becomes more loaded, redder at the tip and edges, and also drier. High-coloured urine is passed with pain and in small quantities, the pupil is often dilated, soreness is felt on pressure over the liver, stomach, and belly, bleeding by the lancet or leeehes is required. Ice to the head gives great relief. In short, the patient is now labouring under a continued fever not to be distinguished from ordinary fever. A profuse critical perspiration may come on, from the second or third day, and leave the sufferer convalescent; but, much more frequently, the quickness of pulse and heat of skin continue, the tongue becomes brown and parched, the eyes are suffused and drowsy, there is a dull flush with stupor and heaviness about the countenance, much resembling typhus, dark sordes collect about the lips and teeth, sometimes the patient is pale, squalid, and low, with the pulse and heat below natural, but with the typhous stupor, delirium supervenes, and death takes place from the fourth to the eighth day, or even later, in the very individual, too, whom the most assiduous attention had barely saved in the first or cold stage. To give a notion of the importance and danger of cholera fever, a most intelligent physician, Dr. Reimer, of the merchant hospital, informs us, that of twenty cases treated under his own eye, who fell victims to the disease, seven died in the cold stage, and thirteen in the consecutive fever.

The singular malady is only cognizable *with certainty* during its blue or cold period. After reaction has been established, it cannot be distinguished from an ordinary continued fever, except by the shortness and fatality of its course. The greenish or dark, and highly bilious discharges produced in the hot stage, by calomel, are not sufficiently diagnostic, and it is curious that the persons employed about these typhoid cases, when they are attacked, are never seized with ordinary fever, but with a genuine cold, blue cholera; nothing, therefore, is more certain, than that persons may come to the coast of England, apparently labouring under common feverish indisposition, who really and truly are suffering under cholera in the second stage.

The points of difference between the present epidemic and the cholera of India, when the two diseases are closely compared, appear to us to be the following:—

First, The evacuations, both upwards and downwards, seem to have been much more profuse and ungovernable in the Indian than in the present cholera, though the characters of the evacuations are precisely the same.

Secondly, Restoration to health from the cold stage, without passing through consecutive fever of any kind, was by far more frequent in India than here, nor did the consecutive fever there assume a typhoid type.

Thirdly, The proportion of deaths in the cold stage, compared with those in the hot, was far greater in India, according to Dr. Russell's experience, than here.

Fourthly, The number of medical men and hospital attendants attacked with cholera during the present epidemic, in proportion to the whole employed and to the other classes of society, has been beyond all comparison greater here than in India under similar circumstances; twenty-five medical men have been already seized, and nine have died out of two hundred and sixty-four. Four others have died at Cronstadt, out of a very small number residing in that fortress at the time the disease broke out there. Six attendants have been taken ill at a small temporary hospital behind the Aboucoff since we wrote last. It is certain, however, that in some cholera hospitals, favourably circumstanced as to size, ventilation, and space, very few of the attendants have suffered.

Of these facts we are likely to receive accurate statements in answer to the written questions which we have submitted to the medical authorities through the Government here.

Convalescence from Cholera has been rapid and perfect here, as is proved by the following fact. The Minister of the Interior had given orders that all convalescents, civil as well as military, at the General Hospital, should be detained fourteen days. We inspected about two hundred of these détenus some days back, with Sir James Wylie, and found them in excellent health, without a single morbid sequela amongst them.

Relapses are rare in this epidemic, nor have they been often attended with fatal results: hospital servants seem to have been most liable to them. One physician had three attacks, the second severe, in which he states that he derived great benefit from the Magisterium bismuthi."

The pamphlet concludes with a short sanatory advice to the inhabitants of towns and villages on the coast, in the event of the cholera appearing in those places. The directions do not differ from those which would be issued by a Quarantine Board, if plague were on the opposite coasts. These directions are familiar to medical readers, and need no notice in this place.

It appears that the Russian Government has abolished all quarantine; but we can scarcely believe that the reason assigned is, "that the whole empire being infected, nothing remains to be gained by the restrictions." Surely no Government in its senses would issue such an astounding annunciation! One thing is clear from this abolition — namely, that quarantine has been of no use in Russia. It will probably turn out that the same measure will be useless to all but those employed in its execution, in more countries than Russia.

